# A Method to Correct the Temporal Drift of Single-Photon Detectors Based on Asynchronous Quantum Ghost Imaging

**DOI:** 10.3390/s24082578

**Published:** 2024-04-18

**Authors:** Carsten Pitsch, Dominik Walter, Leonardo Gasparini, Helge Bürsing, Marc Eichhorn

**Affiliations:** 1 Fraunhofer Institute of Optronics, System Technologies and Image Exploitation (IOSB), Gutleuthausstr. 1, 76275 Ettlingen, Germany; 2 Fondazione Bruno Kessler, Center for Sensors & Devices, Integrated Readout ASICs & Image Sensors, Via Sommarive 18-Povo, 38123 Trento, Italy; 3 Institute of Control Systems (IRS), Karlsruhe Institute of Technology, Fritz-Haber-Weg 1, 76131 Karlsruhe, Germany

**Keywords:** light detection and ranging (LiDAR), single-photon avalanche diode (SPAD), spontaneous parametric down-conversion (SPDC), time-to-digital converter (TDC), temporal drift, quantum ghost imaging (QGI), single-photon timing

## Abstract

Single-photon detection and timing has attracted increasing interest in recent years due to their necessity in the field of quantum sensing and the advantages of single-quanta detection in the field of low-level light imaging. While simple bucket detectors are mature enough for commercial applications, more complex imaging detectors are still a field of research comprising mostly prototype-level detectors. A major problem in these detectors is the implementation of in-pixel timing circuitry, especially for two-dimensional imagers. One of the most promising approaches is the use of voltage-controlled ring resonators in every pixel. Each of these runs independently based on a voltage supplied by a global reference. However, this yields the problem that the supply voltage can change across the chip which, in turn, changes the period of the ring resonator. Due to additional parasitic effects, this problem can worsen with increasing measurement time, leading to drift in the timing information. We present here a method to identify and correct such temporal drifts in single-photon detectors based on asynchronous quantum ghost imaging. We also show the effect of this correction on recent quantum ghost imaging (QGI) measurement from our group.

## 1. Introduction

Single-photon detectors have advanced significantly in recent years due to their necessity in both low-level-light and quantum applications. Current single-photon avalanche diode (SPAD) cameras, based on photon counting, already achieve resolutions in the megapixel regime. However, for many applications, such as ToF-LiDAR [1,2,3,4,5,6], fluorescence lifetime imaging (FLIM) [7,8,9,10], biophotonics [11,12,13,14], and quantum sensing [15,16].

To realize such detectors, SPAD cameras can be outfitted with dedicated timing circuitry in each pixel/group of pixels. These circuits, called time-to-digital converters (TDCs), allow for the registration of the time-of-arrival (ToA) of a single photon per TDC and frame, typically with a resolution of 10 s to 100 s of ps [17,18]. They can be realized in a variety of designs [19,20] and usually return the ToA as an 8 to 12 bit value referenced to the end of the measurement frame. While each design has its dedicated issues, keeping the timing information consistent is a challenge for each.

For 2D detectors, TDC design proves especially challenging as they have to be integrated into the pixel. Commonly used delay-line TDCs [19], which are based on oversampling a global clock signal, are usually very large compared to the size of the SPAD and lead to a highly reduced detector fill factor. One option to reduce TDC size is to use independent TDCs, i.e., a design based on a ring resonator architecture controlled by a common supply voltage. Such structures are, of course, susceptible to instabilities in the supply voltage, leading to a change in the timing period represented by the TDC value. This can prove to be a major limitation as it usually leads to either jitter in the recording or falsified information, i.e., in ToF-LiDAR applications.

This problem is well known, and different experimental setups have been used in the past to identify and address such nonlinearities of TDCs, using experimental measurement techniques [7,21], hardware design [22], or post-processing techniques [23,24]. Using these techniques, the drift in timing information can be calibrated, depending on the nonlinear effects experienced by the TDC.

In this work, we demonstrate a method to experimentally determine the nonlinearities of pixel-level-TDCs by using an asynchronous quantum ghost imaging scheme first demonstrated in [16]. Using this setup, we were able to analyze and correct the temporal drift of a 2D SPAD array used which was outfitted with ring resonator-based TDCs in every pixel. This allowed for single-photon detection with a ~210 ps temporal resolution [25] while maintaining a high fill factor. The single-pixel TDC layout led to a direct, pixel-dependent correction of the timing information. As the detector only returns the TDC value directly, the dependence of the information on this parameter was the only influence we were able to investigate.

However, neither the nonlinearities nor the correction routine are bound to this hardware. Thus, this correction routine can also be applied to a number of different temporally resolving single-photon detectors. In other TDC architectures, other parameters might be given, i.e., START and STOP bits. The method also allows one to investigate dependencies on these parameters, if they are given (see for example [26]). It is only bound by the deterministic nature of the influence/parameter under investigation and fundamentally limited by the cumulated jitter of the SPAD detector [27], the quenching circuit, and the TDC [23].

## 2. Sources of Temporal Drift

Multiple architectures exist for the in-pixel timestamping of circuits in time-resolved, single-photon image sensors, including analog [28] and digital [29,30] implementations. The latter ones are typically based on ring oscillators and can be divided into two main groups: one based on a global ring, whose phases are distributed across the entire array [31,32], and one with replicas of a ring oscillator in every pixel [33]. With a local ring, power consumption is optimized thanks to the reversed start–stop operation. This way, only pixels that detect a photon (typically a small fraction of the entire array) draw current from the supply, while area compactness is achieved using a small technology node [22], or by sacrificing some resolution [25,34].

**Figure 1:**
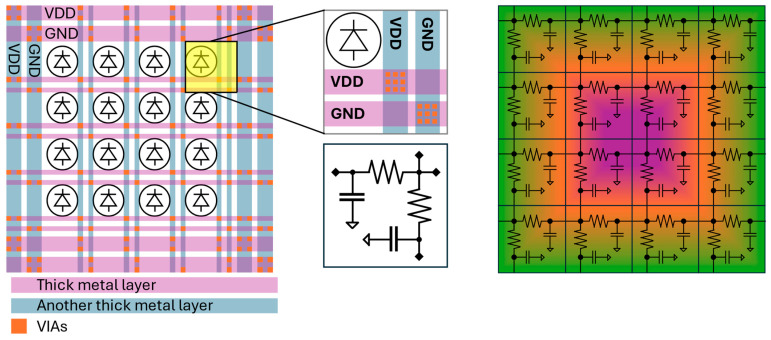
A simplified layout of the power distribution network in a pixel array (**left**), w, with pixel zoom-in (**center**, **top**), and an RC model of a pixel (**center**, **bottom**). The resulting network leads to a non-uniform IR drop which reaches a maximum at the center of the array (**right**).

**Figure 2:**
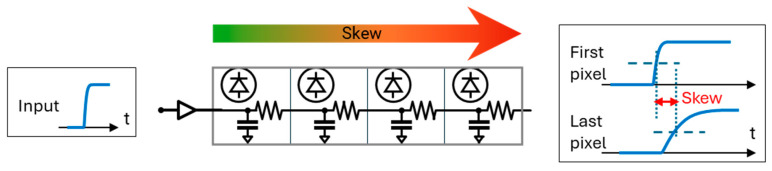
Concept of skew caused by the propagation of the timing signal from the periphery of the array to the pixels.

In theory, photons detected at the same time in different pixels of the detector should return the same timestamp. In reality, the timing information from different pixels will exhibit (usually small) variations from non-idealities, resulting in (slightly) different timing information for every pixel/TDC. Many effects can influence this mismatch, such as jitter, a drop in supply voltage due to the large power consumption of the TDCs (Figure 1), mismatches in the fabrication of the timestamping channels, and skew in the distribution of the common reference signal(s) (Figure 2). While jitter is a random process which can only be analyzed statistically, the influence of the other parameters can be deterministic and can therefore be addressed in the detector’s design or its post-processing.

The power distribution network affects the uniformity of the oscillators due to the voltage drop that occurs when the ring oscillators of the TDC are running. This drop is caused by the current I, which flows in the metal connections of the TDC with a non-zero resistance R. Thus, it is also referred to as an “IR drop”. Figure 1 shows a pictorial representation of the power distribution network of a SPAD imager and the associated IR drop. Typically, a ring of power supply (VDD) and ground (GND) pairs surround the pixel array, distributed horizontally and/or vertically to all pixels to avoid routing them over the active area.

Each pixel contributes to the resistive and capacitive load according to the employed technology, the metal layers used, and the design layout. This increases the voltage drop further down the line, reducing the supply voltage and/or increasing the ground-level voltage the TDC is operated with. This effect is inversely proportional to the length of the transmission line and thus more intense toward the center of the array. The effects can be mitigated by using a regulating transistor for the VDD, but grows worse with an increasing number of TDCs running in parallel. Thus, it poses a limit on the maximum number of detectable photons per observation window.

The manufacturing process causes small differences between the transistor layout and the fabricated counterpart, which introduce ring-to-ring mismatches in the frequency of the oscillation and non-linearities in the TDC transfer function. These can be mitigated in the design phase (i.e., by using a non-minimum transistor size) and calibrated in post-processing since the behavior of a specific TDC is determined during fabrication and remains unchanged during operation.

Another source of non-ideality is the distribution of the reference timing signal which, despite being a global signal, is routed from the periphery to the array. This leads to a delay between pixels, also known as skew (see Figure 2). It can be analyzed by modeling the distribution of the control signal as a transmission line consisting of the resistive and capacitive load of each pixel. The resistance in this model is caused by the metal connections themselves, while the capacitance includes the parasitic effects of the gates and the metal-to-metal/metal-to-substrate connections. These parasitic effects lead to a variation in the control signal, smearing its edges toward later pixels. As the receiver logic is based on edge detection, this leads to a shift in the detection of the control signal, as depicted in Figure 2.

To mitigate this, the overall resistance–capacitance (RC) needs to be minimized. While the capacitance C is typically dominated by the transistor gates and thus cannot be reduced significantly, the resistance R is determined by the width of the metal connections. However, these connections cannot be made arbitrarily wide as they also impact detector performance. In order to achieve a high fill factor and a small pixel pitch, which are required for scalability to large arrays, the lines should be as narrow as possible. Considering both effects in the design phase leads to an optimum width of the connections depending on the design of the array and the envisioned application.

**Figure 3:**
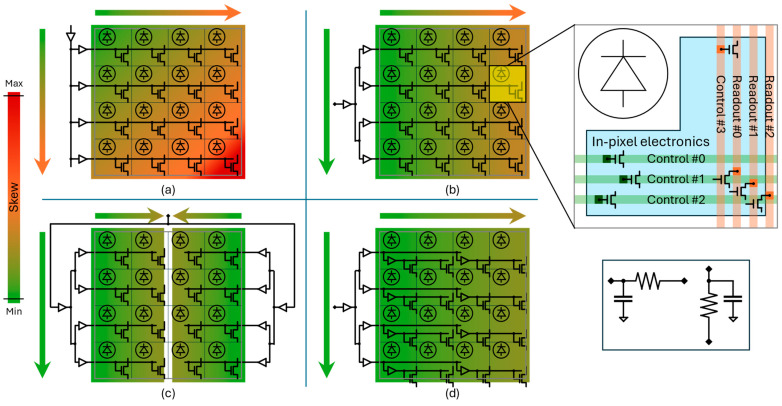
Pictorial representation of the distribution of control signals across the array and associated skew across the array. The worst-case skew depends on the architecture of the driving logic. Here, four common architectures are shown: (**a**) a basic row driver, (**b**) an equalized, row-level buffer tree, (**c**) left and right drivers, and (**d**) an array-level buffer tree. On the right, a pixel zoom-in (**top**) is shown, highlighting the loads of control and readout nets along with the associated RC model (**bottom**).

The skew can be further reduced by properly designing the architecture of the driving logic, as shown in Figure 3. Even for small array sizes (meaning low numbers of TDCs), a single buffer cannot provide enough driving power. Thus, the most simple configuration includes a two-level buffer configuration in which a main buffer drives a set of row-level buffers. In this case, the skew shows a vertical gradient due to the distribution of signal from the main buffer to the row buffers and a horizontal gradient due to the row buffers driving the pixels.

The vertical gradient can be strongly mitigated by using a buffer tree to distribute the signal to the row buffers at the cost of higher complexity and increased power consumption. If the horizontal skew is still too large, the driver can be duplicated on the opposite side of the array so that each driver serves half of the pixels. Alternatively, the tree configuration can be extended inside the pixel array. In this way, the row buffers drive a sub-set of the pixels in each row, and the pixels regenerate the signal and redistribute it locally.

## 3. Correction Approach

In order to measure the temporal drift of the detector under investigation, we exploit the temporal correlation of photon pairs created by spontaneous parametric down-conversion (SPDC) arising from their simultaneous creation [35]. This correlation is usually used in quantum applications, like quantum ghost imaging [15,36], in order to identify photons pairs by so-called coincidence detection.

In our approach, we use a novel setup for quantum ghost imaging (QGI) based on asynchronous detection [16]. This setup allows us to project the detections of two remote detectors in the same time base, which, in turn, allows us to reference their detections to each other. In contrast to QGI, we do not use the spatial correlation of photons to perform imaging but solely the temporal correlation of photons to obtain a timing reference for the detections of the SPAD array. This allows for an investigation of the timing characteristics of a SPAD array by a coincidence analysis.

We are further able to store all relevant data returned by the array for every detection, which allows us to filter the measurement data by specific parameters and investigate their effects on the temporal information of the coincidence analysis. This allows for a detailed analysis of every parameter and parameter combination of the detections of the SPAD camera, allowing us to investigate any deterministic dependency.

### 3.1. Measurement System

**Figure 4:**
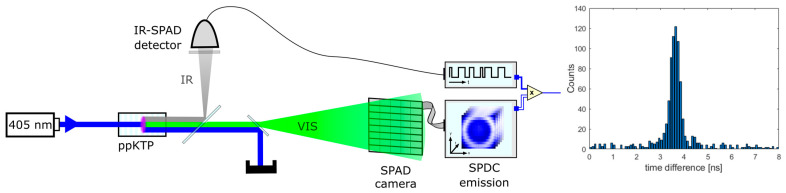
The measurement setup to determine the pixel-dependent temporal drift of a SPAD imager [25]. The setup is based on previously published setups for asynchronous quantum ghost imaging [16]. The emission spot on the camera should illuminate the complete detector aperture in order to perform a coincidence analysis over the whole chip. This should also be verified for the idler detector, especially in case of fiber coupling.

The measurement system is an adapted version of the QGI system described in [16] and shown in Figure 4. The data of the measurements presented in [16] are also used in Section 4 to show the improvement in coincidence detection by correcting the camera’s timing.

As a photon pair source, a 2 mm long, periodically poled potassium titanyl phosphate (KTP) crystal, which is pumped by a 405 nm laser, is used. The crystal’s poling period of 4.25 µm allows for collinear phase matching for non-degenerate photon pairs at 550 nm (signal) and 1550 nm (idler), allowing for the use of readily available, low-jitter infrared SPADs as a timing reference (ID230, [37]). For synchronization, a high-resolution single-photon counting (TCSPC) board (TimeHarp 260 PICO, [38]) is connected to both detectors to enable timestamping every detection with ps resolution.

The optical system is optimized such that the signal emission is distributed over the entire array and the idler emission is optimized for incoupling into the fiber-bound idler detector. This way, coincidence detection is enabled over the whole aperture of the array, which is verified by a preliminary QGI measurement. In particular, due to the fiber-coupling of the idler detector, the assumption of coincidence detection over the whole array is not appropriate from the distribution of the signal emission over the aperture alone is not appropriate.

To ensure the reproducibility of results, more than one measurement should be performed, with their relevant data being compounded for further analysis. Since the amount of processible data is currently limited by the RAM of the system, and significantly more data must be recorded for evaluation, this was already ensured by the automatic restarting of the measurement after a given time interval.

### 3.2. Drift Determination

**Figure 5:**
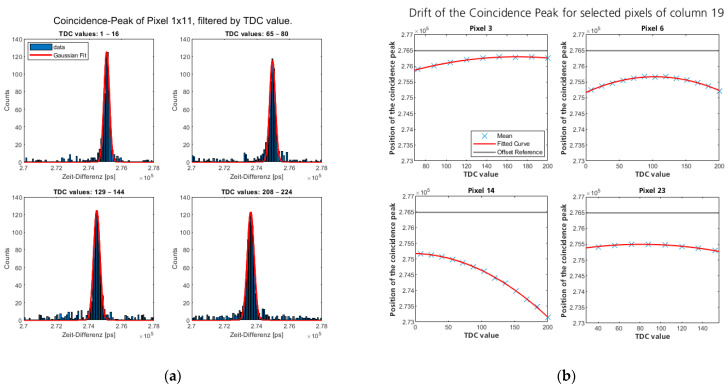
Drift in the coincidence peak over measurement time for selected pixels. (**a**) Coincidence evaluation for groups of TDC values of single pixel (Column 1, Row 11): 16 TDC values were grouped together to ensure enough data for good fit function. Dependence of peak position on TDC value is clearly visible and results in broadening of the peak shown in Figure 6a. (**b**) Evolution of mean of Gaussian fit over TDC values for selected pixels. The drift was estimated by a second-degree polynomial function. To avoid inaccuracies in the fit, only TDC values with enough data were evaluated. Because of this, pixel 23 is only evaluated until the TDC value reaches 160. Also shown is the global reference value used to create the lookup table for the correction.

By comparing the timestamps of both signal and idler detections, a coincidence analysis is enabled. This analysis can be filtered by each parameter individually or specific parameter combinations. In particular, it allows one to filter the detections by both pixel position and the detected ToA given by the TDC value, as shown in Figure 5.

This allows one to analyze differences in both the temporal base resolution of each pixel’s TDC (resulting in “linear drift”) and its behavior over the measurement time (resulting in “nonlinear drift”, see Figure 5). In the case that more information is provided (e.g., START/STOP codes for fine resolution, as in [26]), the influence of these parameters can also be analyzed.

As the uncertainty values in the correlation of the photons and the timing jitter of the reference detector (~150 ps) are smaller than the resolution of the detector under investigation (~210 ps resolution), the coincidence evaluation allows us to determine any influence of the available parameters on the temporal behavior of the detection. It allows this analysis not just in terms of drift in the timing information, but also e.g., in timing jitter by the investigation of the peak broadening. For such an analysis, however, the jitter of the reference detector should be decreased further to reduce its influence.

## 4. Results

**Figure 6:**
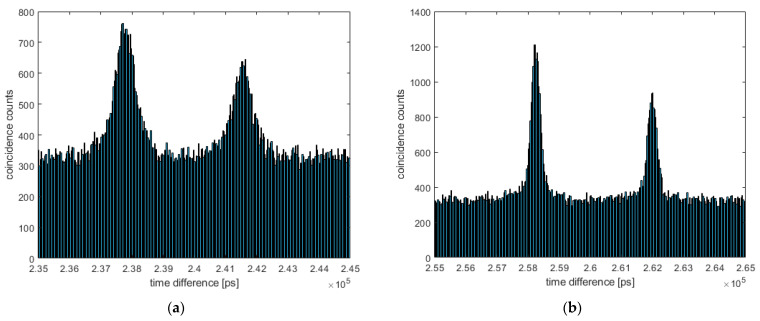
A coincidence evaluation of the Michelson measurement of [16] with different corrections. (**a**) A coincidence peak with the linear correction of the TDC values, as published in [16]. The peak is about 2 ns wide, with an FWHM of about 700 ps. (**b**) The coincidence peak with the proposed drift correction, yielding a full peak width of about 700 ps and an FWHM of about 300 ps.

**Figure 7:**
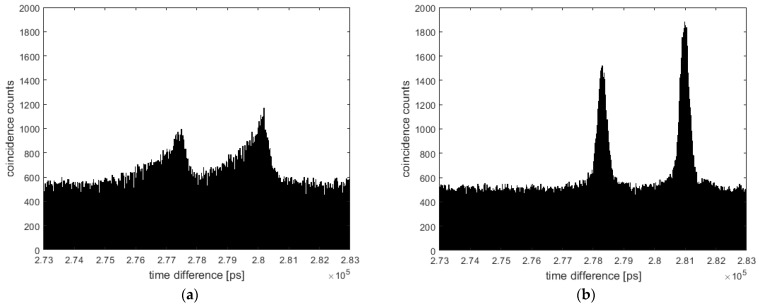
A coincidence evaluation of the free space measurement of [16] with different corrections. (**a**) The coincidence peak with a coarse linear correction of the TDC values detailed in [25] (not shown in [16]). The peak is about 3 ns wide with an FWHM of about 700 ps and a considerable “tail”. (**b**) A coincidence peak showing the proposed drift correction, yielding a full peak width of about 700 ps and an FWHM of about 300 ps.

In order to test the approach and validate the improvement, we performed the correction on recently published measurements detailed in [16]. The resulting coincidence peaks and improvements are shown in Figure 6 and Figure 7.

To do so, we built the measurement setup shown in Figure 4, operated it with a 20 mW pulsed laser as pump, and investigated the detector used in [16]. In order to obtain enough data for a detailed analysis, we performed continuous measurements over 4 days. Due to limitations in the processible amount of data, individual measurements were limited to 2 h and then automatically restarted.

For further processing, each of the measurements was evaluated individually, filtered by pixels. This isolated the individual TDCs associated with each pixel, which showed individual timing drifts (see Section 2). The temporal behavior of the TDCs was then analyzed by filtering each pixel’s data, this time by the value of the TDC itself (corresponding to the ToA of the photon). To ensure enough data yield for a meaningful analysis, the TDCs (8 bit = 256 values) were grouped into 16 groups with 16 TDC values each. The processed data were then stored and compounded with all other measurements for further analysis.

In order to allow for the evaluation of the complete set of measurements, the data of the coincidence evaluation had to be reduced. To do so, a 5 ns wide coincidence window was defined around the peak determined by the preliminary QGI measurement used for calibration (see Section 3.1). This window was split into sections 100 ps large for which a histogram analysis of the coincidence counts was performed. The results where then saved as a (32 × 32 × 16 × 50) array (*Row × Column × TDC group × Coincidence window*), allowing us to simply add up the coincidence counts of each measurement, increasing the amount of data and thus the validity of the drift estimation.

The evolution of the coincidence peak over the TDC value is exemplary, as shown in Figure 5a for a single pixel and selected TDC values. With the data shown, the coincidence peak is analyzed by fitting a normal Gaussian distribution to the coincidence distribution. The mean of this fit is then used to determine the drift of each pixel’s timing information, as shown exemplarily in Figure 5b for selected pixels.

Due to the detector’s architecture (see [25]), the highest TDC values (~above 200) are unlikely to occur, resulting in too few data for valid analysis. To avoid problems, the TDC groups were filtered by the amount of overall data before they were considered for the estimation of the temporal drift. Thus, the evaluation shown in Figure 5b shows different limits on its x scale, none of which are above a TDC value of 200.

The drift was estimated by fitting a polynomial function of the second order to the data obtained and projecting said estimation to all possible TDC values (256 values). In an initial correction approach, the parameters of the fit were passed to the evaluation code of the original dataset of [16], calculating the offset for each detection. However, this approach highly increased the runtime of the code for the coincidence evaluation and thus was not fit for usage. Instead, the offset of the coincidence peak (mean) to a global reference value was calculated for every possible TDC value. With these values, a lookup table was created. For every detection, we then added the dedicated lookup value (determined by the pixel number and TDC value) to our detection time, thus eliminating the drift.

Applying this correction to the measurement of [16], the coincidence peak could be improved from a full width of about 2 ns (FWHM: 700 ps) with a linear correction to about 700 ps (FWHM: 300 ps), as shown in Figure 6. This peak width already comes close to the resolution of the detector’s TDCs of ~210 ps [25]. We presume this peak width is currently also limited by the resolution of the reference detector in the idler, which shows a jitter of ~150 ps [37], and the TDC resolution used for the correction (groups of 16 values). It should be mentioned that the linear correction in [16] was already refined using coincidence data. The drift correction, however, was based on a coarse linear correction detailed in [25]. The coincidence peak achieved with this coarse correction is shown in Figure 7 for the free space measurement of [16], resulting in a peak width of about 3 ns and significant “tail”. Both the peak width and tail could be highly improved using the proposed correction/calibration method.

## 5. Outlook

We have shown here a method to accurately determine the temporal behavior of single-photon cameras, exploiting the temporal correlation of photon pairs created by SPDC.

The correction of this temporal drift allowed us to increase the temporal resolution of the coincidence analysis of a recent asynchronous QGI measurement by a factor of ~3. The achieved resolution of a ~700 ps full peak width is already close to the base resolution of the detector under investigation (~210 ps) and the reference detector used (~150 ps). It might be improved further by a more detailed analysis of TDC dependence, especially by increasing the resolution of the drift determination, optimally by analyzing each individual TDC value instead of groups of 16 values. For this analysis to be valid, however, more data should be acquired.

Another improvement would be the use of a more precise bucket detector, i.e., a super-conducting nanowire single-photon detector (SNSPD). Using a low-jitter silicon detector as a bucket might also be an option, but for most SPAD imagers, the wavelength of the signal photons should be kept in the 550 nm regime due to the detection efficiency of the pixels. The realization of SPDC with this signal wavelength and an idler wavelength in the silicon detection range does lead to problems regarding the photon-pair source. Due to energy conservation, a UV laser would need to be used, leading to problems regarding the degradation of the nonlinear crystal.

However, the system is not limited by the use of SPDC but only the fundamental temporal correlation of the photons. Thus, any source of time-correlated photon states (i.e., also four-wave mixing) could be used. The correlation must only be verified to be more exact than the resolution of the detectors.

Compared to other experimental calibration techniques, like temporally shifted lasers [7], this method allows for the calibration of every pixel’s TDC with potentially lower uncertainty. As for a laser-based scheme, the emission and detection usually underly Poissonian photon statistics, reducing the achievable timing resolution of the analysis.

For a good correction, fast switching and relatively powerful lasers would typically be needed to reduce this effect. The resulting flood illumination can, however, impact detector performance, especially the timing properties under investigation, leading, e.g., to voltage instabilities in the TDCs. This would, in turn, falsify the temporal information and correction for key low-level light applications.

Our approach, however, works with illumination levels similar to other quantum and low-level-light applications, which ensures the same detection characteristics.

## Data Availability

Data are contained within the article.
